# Tumor Necrosis Factor Receptor-Associated Factor 5 Interacts with the NS3 Protein and Promotes Classical Swine Fever Virus Replication

**DOI:** 10.3390/v10060305

**Published:** 2018-06-05

**Authors:** Huifang Lv, Wang Dong, Kangkang Guo, Mingxing Jin, Xiaomeng Li, Cunfa Li, Yanming Zhang

**Affiliations:** 1 College of Veterinary Medicine, Northwest A&F University, Yangling 712100, China; lvhuifang123@126.com (H.L.); superdong1990@163.com (W.D.); guokk2007@nwafu.edu.cn (K.G.); 15691972175@163.com (M.J.); 2 College of Pharmaceutical Engineering, Henan University of Animal Husbandry and Economy, Zhengzhou 450046, China; licunfa@126.com; 3 College of Veterinary Medicine, Henan University of Animal Husbandry and Economy, Zhengzhou 450046, China; 4 Ningbo Entry-Exit Inspection and Quarantine Bureau, Ningbo 315000, China; lxm1154600411@163.com

**Keywords:** CSFV, NS3, p38 MAPK, TRAF5, replication

## Abstract

Classical swine fever, caused by classical swine fever virus (CSFV), is a highly contagious and high-mortality viral disease, causing huge economic losses in the swine industry worldwide. CSFV non-structural protein 3 (NS3), a multifunctional protein, plays crucial roles in viral replication. However, how NS3 exactly exerts these functions is currently unknown. Here, we identified tumor necrosis factor receptor-associated factor 5 (TRAF5) as a novel binding partner of the NS3 protein via yeast two-hybrid, co-immunoprecipitation and glutathione *S*-transferase pull-down assays. Furthermore, we observed that TRAF5 promoted CSFV replication in porcine alveolar macrophages (PAMs). Additionally, CSFV infection or NS3 expression upregulated TRAF5 expression, implying that CSFV may exploit TRAF5 via NS3 for better growth. Moreover, CSFV infection and TRAF5 expression activated p38 mitogen activated protein kinase (MAPK) activity, and inhibition of p38 MAPK activation by the SB203580 inhibitor suppressed CSFV replication. Notably, TRAF5 overexpression did not promote CSFV replication following inhibition of p38 MAPK activation. Our findings reveal that TRAF5 promotes CSFV replication via p38 MAPK activation. This work provides a novel insight into the role of TRAF5 in CSFV replication capacity.

## 1. Introduction

Classical swine fever virus (CSFV) is the etiological agent of classical swine fever, which is a highly contagious and high-mortality viral disease, causing huge economic losses in the swine industry worldwide. CSFV is a single-stranded, positive-sense RNA virus, within the genus *Pestivirus* of the family *Flaviviridae* [1]. The 12.3-kb genome of CSFV encodes a single polyprotein, which is further processed into 12 mature proteins, including four structural proteins (C, E^rns^, E1 and E2) and eight non-structural (NS) proteins (N^pro^, p7, NS2, NS3, NS4A, NS4B, NS5A and NS5B) [2]. CSFV NS3 is a multifunctional non-structural protein, which plays an essential role in viral replication. NS3 possesses helicase, nucleotide triphosphatase (NTPase) and serine protease activities [3,4,5]. NS3 interacts with NS5B and enhances RNA-dependent RNA polymerase activity of NS5B [6,7]. NS3 is also an internal ribosome-entry site (IRES)-binding protein that increases IRES-dependent translation [8]. Additionally, NS3 expression results in the cytopathic effect of PK-15 cells [9].

In our previous study, we screened 26 cellular partners interacting with NS3 by the yeast two-hybrid (Y2H) system, including tumor necrosis factor (TNF) receptor-associated factor (TRAF) 5 and TRAF6. In addition, we showed that TRAF6 inhibits CSFV replication by activating the NF-κB signaling pathway [10]. TRAFs, cytoplasmic adaptor proteins, link cell-surface receptors to intracellular signaling pathways that play major roles in many biological processes, such as immune regulation, inflammatory responses and apoptosis [11,12]. TRAF5, is composed of an N-terminal Really Interesting New Gene (RING) finger domain, a zinc finger motif, leucine-zipper domain (TRAF-N) and a C-terminal receptor binding domain (TRAF-C) [13,14]. TRAF5, the downstream target of MAVS, mediates the activation of downstream nuclear factor-kappa B (NF-κB), interferon regulatory factor 3 (IRF3) and mitogen activated protein kinase (MAPK) signaling pathways [15,16,17].

MAPK, a serine/threonine kinase, is an important molecular that receives receptor signal and transfers it into the nucleus. It plays a key role in gene expression regulation, cell proliferation and cell death [18,19,20]. p38 MAPK, one of the MAPK family, is mainly involved in immune regulation, inflammatory response and apoptosis under stress conditions [21,22,23]. The p38 MAPK signaling pathway also participates in macrophage and neutrophil functional response, including chemotaxis and adhesion [24,25]. It has been reported that TRAF5 is involved in the regulation of MAPK phosphorylation in the melanoma A375 and B16F10 cells [26]. TRAF5 engages in glucocorticoid-induced tumor necrosis factor receptor (GITR)-induced activation of p38 MAPK [27]. In addition, TRAF5 expression induces p38 MAPK activation in HeLa cells [28]. TRAF5 activates p38 MAPK to induce human immunodeficiency virus 1 (HIV-1) gene expression in monocytes/macrophages [29]. Activation of TRAF5 negatively regulates the latent replication origin of epstein-barr virus through p38 MAPK [28].

TRAF5 mediates the activation of the p38 MAPK signaling pathway, and we have found that TRAF5 is also a potential binding partner of CSFV NS3 and that TRAF6 is shown to be relevant for CSFV infection. Thus, we hypothesise that TRAF5 may influence CSFV replication through p38 MAPK activation. Here, we demonstrated that CSFV NS3 interacted with TRAF5 and promoted TRAF5 expression, which in turn promoted CSFV replication via activating p38 MAPK.

## 2. Materials and Methods

### 2.1. Cells and Virus

Porcine alveolar macrophages (PAMs) (American Type Culture Collection; CRL-2845) were cultured in Roswell Park Memorial Institute (RPMI) 1640 medium (Gibco, Grand Island, UK). Human embryonic kidney (HEK293T; ATCC; CRL-11268) cells were cultured in Dulbecco’s minimal essential medium (DMEM) (Gibco, UK) with 10% fetal bovine serum (FBS) (Biowest, Loire Valley, France). CSFV (Shimen strain) was purchased from the Control Institute of Veterinary Bio-products and Pharmaceuticals (Beijing, China). CSFV titers in culture supernatant were determined by indirect immunofluorescence assay (IFA) as described previously [10,30].

### 2.2. Plasmid Construction

CSFV NS3 was amplified by polymerase chain reaction (PCR) and cloned into pGBKT7 (BD), pCDH-CMV-MCS-EF1 with a Flag-tag, pEGFP-N1 and pGEX-6P-1 to generate BD-NS3, CMV-NS3/NS3-Flag, NS3-GFP and GST-NS3, respectively. Red fluorescent protein (RFP) gene was amplified and cloned into pCDH-CMV-MCS-EF1 with a Flag-tag to generate RFP-Flag. These vectors were constructed and preserved in our laboratory [10]. The full-length TRAF5 was amplified by polymerase chain reaction (PCR) from PAMs cDNA and cloned into pGADT7 (AD), pGEX-6P-1 and pCDH-CMV-MCS-EF1 with a Flag-tag to generate AD-TRAF5, GST-TRAF5 and CMV-TRAF5/TRAF5-Flag, respectively. Three pairs of shRNAs targeting TRAF5 were designed (http://rnaidesigner.thermofisher.com/), and the annealing fragments were cloned into pCDH-U6-MCS-EF1-GreenPuro to create TRAF5-sh1, TRAF5-sh2, TRAF5-sh3, respectively. Negative control vector shN was conserved in our laboratory [10]. All primers are listed in Table 1.

### 2.3. Real-Time Quantitative PCR (RT-qRCR)

The relative mRNA expression of TRAF5 and CSFV genome RNA was tested by RT-qPCR using specific primers (Table 1). Total cellular RNA was extracted from PAMs using TRIzol, and the cDNA was synthesized by reverse transcription with the PrimeScript RT reagent kit (Vazyme, Nanjing, China). Gene expression was quantified via a Bio-Rad iQ5 Multicolor Real-Time PCR Detection System with GoTaq^®^ Master Mix (Promega, Madison, WI, USA) according to the manufacturer’s protocol. The housekeeping gene, β-actin, served to normalize the relative expression of each gene. Relative transcript levels were analyzed using the ΔΔ*C*t method with β-actin as the internal control by the manufacturer [31].

### 2.4. Western Blot

Protein samples were separated by 12% SDS-PAGE and were transferred onto polyvinylidene difluoride (PVDF) membranes (catalog no. ISEQ00010; Millipore, Burlington, MA, USA). The membranes were blocked with 5% skim milk at room temperature for 2 h, followed by incubation with indicated primary antibodies at room temperature for 2 h; for example, rabbit anti-TRAF5 polyclonal antibody (pAb) (catalog no. orb186283; Biorbyt, Cambridgeshire, UK), rabbit anti-MAPK14 (p38 MAPK) pAb (catalog no. CSB-PA003661; Cusabio, Wuhan, China), rabbit anti-phospho-MAPK14 (T180/Y182) pAb (catalog no. CSB-PA000647; Cusabio, China), mouse anti-GFP monoclonal antibody (mAb) (catalog no. KM8009; SUNGENE BIOTECH, Tianjin, China), mouse anti-Flag mAb (catalog no. CW0287; CWBIO, Beijing, China), and mouse anti-GST mAb (catalog no. CW0291; CWBIO, China). After incubation with horseradish peroxidase-conjugated goat anti-rabbit (or mouse) IgG secondary antibody (catalog no. LK2001 or LK2003; SUNGENE BIOTECH, China), the signal was visualized using an image analysis system (Bio-Rad, Hercules, CA, USA).

### 2.5. Yeast Two-Hybrid Screening

To confirm the interaction between NS3 and TRAF5 by yeast two-hybrid assay, a Matchmaker Gold Yeast Two-Hybrid system (Clontech, Fremont, CA, USA) was applied as described previously [30]. Y2HGold was cotransformed with BD-NS3 or BD and the full-length TRAF5 (AD-TRAF5). The transformants were plated on double-dropout plates lacking Leu and Trp (DDO) and quadruple-dropout plates lacking His, Leu, Trp, Ade (QDO) and QDO containing X-alpha-galactosidase and aureobasidin A (QDO/X/Aba) plates. Cotransformations with BD/AD, BD-Lam (human lamin C protein)/AD-T and BD-p53/AD-T (simian virus 40 large T antigen) served as blank, negative and positive controls, respectively.

### 2.6. Co-Immunoprecipitation Assays

For co-IP assays, exogenous expression and endogenous verification were performed. For exogenous verification, PAMs in 6-well pates (10^5^ cells) were co-transfected with 2 μg of NS3-GFP and 2 μg of TRAF5-Flag plasmids, co-transfection with NS3-GFP and RFP-Flag as negative control. The 20% of cell lysates were subjected to an input experiment, and the immunoprecipitation experiments were carried out with ANTI-FLAG M2 Affinity Gel (Sigma, St. Louis, MO, USA) with the rest of cell lysates according to the manufacturer’s instructions. The washed resins were analyzed by Western blot with a mouse anti-GFP mAb. For endogenous verification, PAMs (10^5^ cells) were transfected with 4 μg of NS3-Flag or RFP-Flag plasmids as control, and the washed resins were analyzed by Western blot with a rabbit anti-TRAF5 pAb.

### 2.7. GST Pull-Down Experiment

A GST pull-down experiment was carried out as described previously [10]. Briefly, GST-NS3 was expressed in *Escherichia coli* (*E. coli*) Rosetta cells, and TRAF5-Flag was expressed in HEK293T cells. The Pierce^®^ GST Protein Interaction Pull-Down Kit (Thermo, Waltham, MA, USA) was used according to the manufacturer’s instructions. Briefly, GST or GST-NS3 protein expressed in *E. coli* was treated with pull-down lysis buffer and immobilized on equilibrated glutathione agarose resin for 2 h at 4 °C. After five washes with a wash solution, the TRAF5-Flag protein in HEK293T lysates was added and incubated overnight at 4 °C. After another five washes, protein samples were eluted with glutathione elution buffer. Western blot detected the eluted proteins with an anti-Flag mAb. Furthermore, GST-TRAF5 expressed in *E. coli* and NS3-Flag expressed in HEK293T cells were also subjected to GST pull-down experiments to further verify the interaction between NS3 and TRAF5 as described above.

### 2.8. Cell Viability Assay

To evaluate the effects of SB203580 (Beyotime, Shanghai, China) on the growth of PAMs, cell viability was measured by the cell counting kit-8 (CCK-8; Beyotime, China) according to the manufacturer’s instructions.

### 2.9. Statistical Analysis

Data are presented as the mean ± standard deviation (SD) of three replicates. The data were analyzed by one-way ANOVA and Bonferroni post-hoc test using the SPSS software (version 18.0, International Business Machines Corporation, NY, USA). A *p* < 0.05 was considered statistically significant.

## 3. Results

### 3.1. CSFV NS3 Interacts with TRAF5

**Figure 1:**
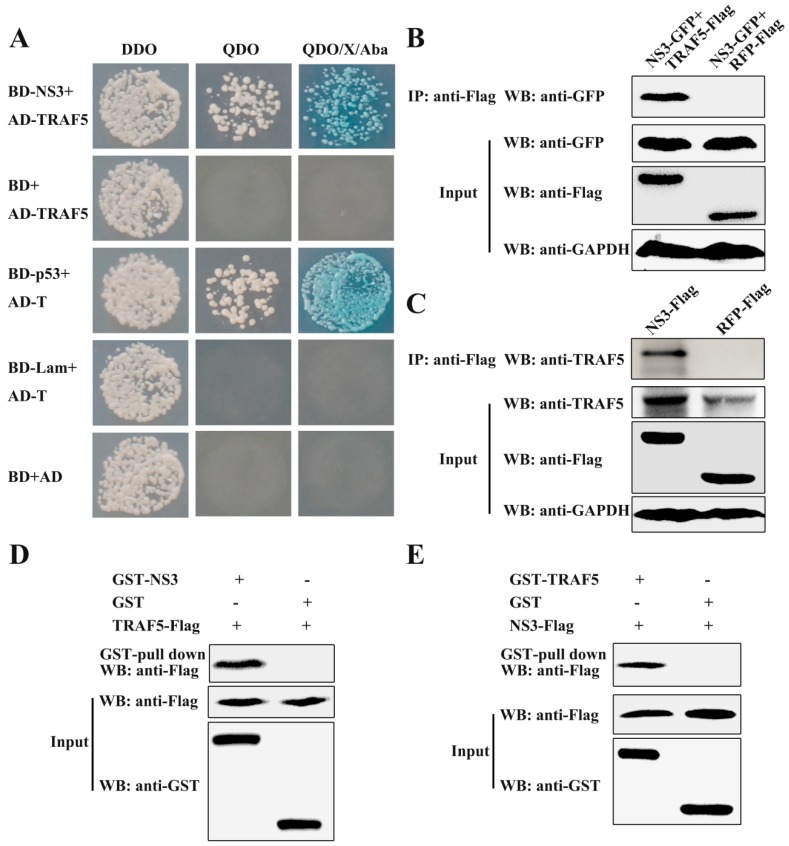
Interaction of non-structural protein 3 (NS3) with tumor necrosis factor receptor-associated factor 5 (TRAF5). (**A**) Interaction of NS3 and TRAF5 by the Y2H system. The yeast strain Y2HGold was cotransformed with the full-length TRAF5 (AD-TRAF5) and the bait plasmid BD or BD-NS3. Cotransformations with BD/AD, BD-Lamin/AD-T and BD-p53/AD-T were used as blank, negative and positive controls, respectively; (**B**) Exogenous co-immunoprecipitation (co-IP) analysis of NS3 and TRAF5 in porcine alveolar macrophages (PAMs). Cells were co-transfected with plasmids NS3-GFP and TRAF5-Flag; co-transfection with NS3-GFP and RFP-Flag as a negative control. In total, 20% of the cell extract was subjected to the input assay to assess GAPDH, Flag-fusion, and GFP-fusion protein levels. The rest of the extract was subjected to IP assay and precipitated proteins were detected by Western blot with a mouse anti-GFP mAb; (**C**) Endogenous co-IP analysis of NS3 and TRAF5 in PAMs. PAMs were transfected with plasmid NS3-Flag; transfection with RFP-Flag as a negative control. The precipitated proteins were detected by western blot with a rabbit anti-TRAF5 pAb; (**D**) GST-NS3 pull-down assay. The glutathione *S*-transferase (GST) and GST-NS3 proteins expressed in *E. coli* Rosetta (DE3) cells were immobilised on a glutathione agarose resin, followed by incubation of the resin with the HEK293T cell lysates containing TRAF5-Flag protein. The bound proteins were detected by Western blot using a mouse anti-Flag mAb. The expression of input proteins (TRAF5-Flag, GST or GST-NS3) was confirmed by Western blot using a mouse anti-Flag mAb and a mouse anti-GST mAb, respectively; (**E**) GST-TRAF5 pull-down assay. The GST and GST-TRAF5 proteins expressed in *E. coli* Rosetta (DE3) cells were immobilised on the glutathione agarose resin, with incubation with the HEK293T cell lysates containing the NS3-Flag protein. The eluted proteins were detected by Western blot using a mouse anti-Flag mAb.

In previous studies, we reported that there are 26 cellular proteins interacting with the CSFV NS3 protein, including TRAF5 [10]. To further explore the function of NS3, TRAF5 was selected to study the relationship between NS3, TRAF5 and CSFV replication. First, the prey vector containing full-length TRAF5 was constructed, and the TRAF5-NS3 interaction was verified by the Y2H system. The yeast strain Y2HGold was cotransformed with the prey plasmid AD-TRAF5 and the bait plasmid BD-NS3 or BD. The results showed that the cotransformations with BD-NS3/AD-TRAF5 and BD-p53/AD-T were grown on the QDO/X/A plates, indicating the interaction of TRAF5 and NS3 (Figure 1A).

To further validate the TRAF5-NS3 interaction, exogenous co-immunoprecipitation (co-IP) experiments were performed by co-expressing NS3-GFP and TRAF5-Flag. After incubation with ANTI-FLAG M2 Affinity Gel, TRAF5-Flag successfully precipitated NS3-GFP (Figure 1B). Endogenous co-IP experiments were also carried out by expressing NS3-Flag, and the result showed that NS3-Flag precipitated endogenous TRAF5 (Figure 1C). Glutathione *S*-transferase (GST) pull-down experiments were carried out for verifying the TRAF5-NS3 interaction in vitro. GST-NS3 expressed in *E. coli* Rosetta cells and TRAF5-Flag expressed in HEK293T cells were subjected to the Pierce spin column immobilised with glutathione agarose. After elution, TRAF5-Flag was captured by GST-NS3 rather than by GST (Figure 1D). Inversely, GST-TRAF5 expressed in *E. coli* Rosetta cells and NS3-Flag protein expressed in HEK293T cells were subjected to the GST pull-down assay. Expectedly, NS3-Flag was captured by GST-TRAF5 rather than by GST (Figure 1E), suggesting the specificity of the TRAF5-NS3 interaction. These results, along with the previous Y2H system and co-IP results, demonstrated the interaction of TRAF5 and NS3.

### 3.2. TRAF5 Knockdown Inhibits CSFV Replication

**Figure 2:**
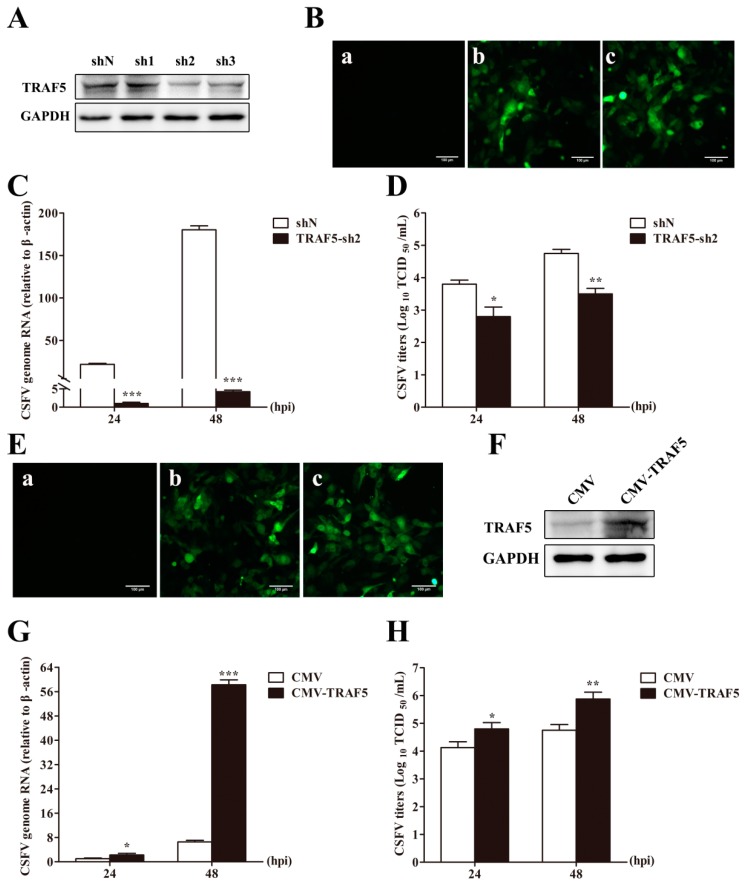
TRAF5 promotes CSFV replication. (**A**) Western blot analysis of knockdown efficiency of three TRAF5 knockdown vectors. PAMs were transfected with TRAF5-sh1, TRAF5-sh2, TRAF5-sh3, or shN. At 48 h post transfection (hpt), the cell lysates were collected and endogenous TRAF5 expression was determined by Western blot. GAPDH was used as an internal control; (**B**) Confirmation of TRAF5-knockdown lentivirus infection by fluorescence detection of the GFP reporter expressed in PAMs. TRAF5-knockdown PAMs were constructed via lentivirus infection. (a) Mock-infected PAMs. (b) PAMs infected with lentiviruses expressing shN. (c) PAMs infected with TRAF5-sh2 lentivirus. Scale bar = 100 μm for all the figures; (**C**) CSFV genome RNA in TRAF5-sh2 cells. The shN and TRAF5-sh2 cells were infected with CSFV at an MOI of 0.1. CSFV genome RNA levels were determined by RT-qPCR at 24 and 48 hpi; (**D**) Viral titers of infectious progeny in the supernatant from TRAF5-sh2 cells. Viral titers in the culture supernatant collected at 24 and 48 hpi were determined and expressed as TCID_50_/mL; (**E**) Confirmation of CMV-TRAF5 recombinant lentivirus infection by fluorescence detection of the GFP reporter expressed in PAMs. (a) Mock-infected PAMs. (b) PAMs infected with lentiviruses expressing CMV. (c) PAMs infected with lentiviruses expressing CMV-TRAF5. Scale bar = 100 μm for all the figures; (**F**) Western blot for TRAF5 expression in CMV-TRAF5 cells; (**G**) CSFV genome RNA in CMV-TRAF5 cells. The CMV and CMV-TRAF5 cells were infected with CSFV at an MOI of 0.1. CSFV genome RNA levels were determined by RT-qPCR at 24 and 48 hpi; (**H**) Infectious progeny viral titers in supernatants from CMV-TRAF5 cells. Viral titers in the culture supernatant collected at 24 and 48 hpi were determined and expressed as TCID_50_/mL. Error bars represent the mean ± SD of three independent experiments. *, *p* < 0.05; **, *p* < 0.01; *** *p* < 0.001.

To detect the relationship between TRAF5 and CSFV replication, we determined CSFV replication in TRAF5 knockdown PAMs. Three shRNA-mediated TRAF5 knockdown vectors were designed and the knockdown efficiency was detected. As shown in Figure 2A, TRAF5-sh2 revealed the highest knockdown efficiency (83.37%). Thus, the knockdown cell line (TRAF5-sh2) targeting TRAF5 was constructed using the TRAF5-sh2 vector via lentivirus infection in PAMs according to the methods described previously [10,30]. The cell line stably expressing shN (shN) was treated equally as a negative control. The green fluorescence in shN and TRAF5-sh2 cells was visible under an inverted fluorescence microscope (Figure 2B). The stable cell lines were infected with CSFV at an MOI of 0.1, and CSFV genome RNA and viral titers of the infectious progeny were determined in cell lysates and culture supernatant, respectively, at 24 and 48 h post infection (hpi). The results demonstrated that CSFV genome RNA levels and viral titers of the infectious progeny in TRAF5-sh2 knockdown cells were significantly decreased at 24 and 48 hpi, respectively, compared to those in shN cells (Figure 2C,D), suggesting that TRAF5 knockdown inhibited CSFV replication.

### 3.3. TRAF5 Overexpression Promotes CSFV Replication

To further verify the effect of TRAF5 on CSFV replication, a cell line stably overexpressing TRAF5 (CMV-TRAF5) was constructed via lentivirus infection in PAMs, with PAMs stably expressing the empty vector CMV (CMV) as a negative control. As shown in Figure 2E, the green fluorescence in CMV and CMV-TRAF5 cells was visible under an inverted fluorescence microscope. Additionally, TRAF5 overexpression was confirmed by Western blot with a rabbit anti-TRAF5 pAb (Figure 2F). Following CSFV infection at an MOI of 0.1, the cell lysates and supernatant were collected to detect CSFV genome RNA and viral titers, respectively. The results showed that CSFV genome RNA and viral titers of the infectious progeny in CMV-TRAF5 cells were significantly increased at 24 and 48 hpi compared with those in CMV cells (Figure 2G,H), indicating that TRAF5 positively regulated CSFV replication in PAMs.

### 3.4. CSFV or NS3 Promotes TRAF5 Expression

**Figure 3:**
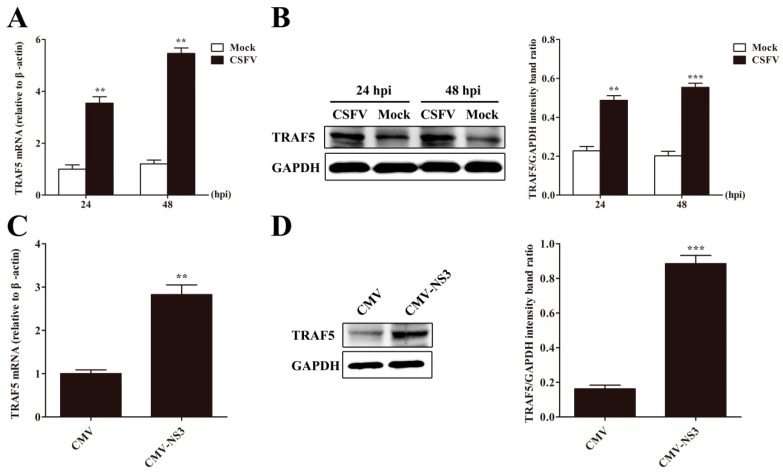
CSFV or NS3 promotes TRAF5 expression. (**A**) TRAF5 mRNA expression in CSFV-infected PAMs. PAMs were mock-infected or CSFV-infected. TRAF5 mRNA was analyzed by RT-qPCR at 24 and 48 hpi; (**B**) TRAF5 protein expression in CSFV-infected PAMs. TRAF5 protein levels were analyzed by Western blot at 24 and 48 hpi. GAPDH served as an internal control. The relative levels of TRAF5 protein were estimated by histograms representing density readings of the gel bands and the ratios were calculated relative to GAPDH control; (**C**) TRAF5 mRNA in NS3-expressing PAMs. PAMs were transfected with CMV-NS3. TRAF5 mRNA expression was analyzed by RT-qPCR at 36 hpt; (**D**) TRAF5 protein expression in NS3-expressing PAM. TRAF5 protein expression was analyzed by Western blot at 36 hpt. The relative levels of the TRAF5 protein were estimated by histograms representing density readings of the gel bands and the ratios were calculated relative to GAPDH control. The data represent the mean ± SD of three independent experiments. **, *p* < 0.01; ***, *p* < 0.001.

After the verification of positive effects of TRAF5 on CSFV replication, we investigated the effect of CSFV infection on TRAF5 expression. TRAF5 mRNA and protein levels were analyzed in CSFV-infected and mock-infected PAMs by RT-qPCR and Western blot at 24 and 48 hpi. We observed that TRAF5 mRNA and protein levels were significantly increased in CSFV-infected PAMs than that in mock-infected cells at 24 and 48 hpi (Figure 3A,B). Because of the interaction of TRAF5 and NS3, the effect of CSFV NS3 on TRAF5 expression was investigated. As shown in Figure 3C,D, TRAF5 mRNA and protein expression was increased in PAMs expressing NS3. These results revealed that CSFV or NS3 increased cellular TRAF5 expression. Together with the fact that TRAF5 promotes CSFV propagation, these data imply that CSFV may exploit TRAF5 via NS3 for better growth and that TRAF5 plays an important role during CSFV infection.

### 3.5. CSFV Infection Promotes p38 MAPK Activation in PAMs

**Figure 4:**
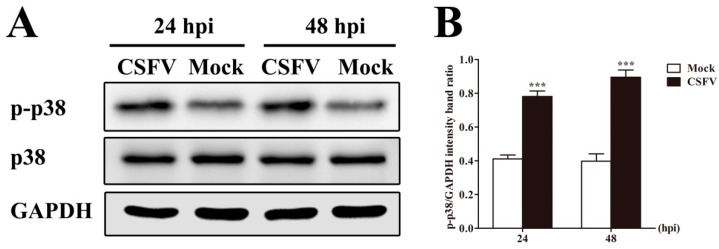
CSFV infection promotes p38 MAPK activation in PAMs. (**A**) Western blot analysis of p-p38 and p38 in CSFV-infected PAMs. PAMs were mock-infected or CSFV-infected at an MOI of 0.1. The cell lysates were collected at 24 and 48 hpi and used to determine p-p38 and p38 by Western blot; (**B**) The relative levels of p-p38 MAPK were estimated by histograms representing density readings of the gel bands and the ratios were calculated relative to GAPDH control. The data represent the mean ± SD of three independent experiments. ***, *p* < 0.001.

We have verified that TRAF5 promoted CSFV propagation and that TRAF5 may play an important role during CSFV infection. It has been reported that TRAF5 induces HIV-1 gene expression via activation of p38 MAPK in monocytes/macrophages [29]. To explore the mechanism of enhancement of CSFV propagation by TRAF5, we first investigated the effect of CSFV on the activation of p38 MAPK. PAMs were mock-infected or CSFV-infected at an MOI of 0.1 and the cell lysates were prepared for detection of total p38 MAPK (p38) and phosphorylated p38 MAPK (p-p38) levels at 24 and 48 hpi. The results showed that CSFV infection increased p38 MAPK phosphorylation and had no effects on total p38 MAPK levels at 24 and 48 hpi (Figure 4A,B). These data suggest that CSFV infection promotes p38 MAPK activation in PAMs.

### 3.6. TRAF5 Promotes p38 MAPK Activation in PAMs

**Figure 5:**
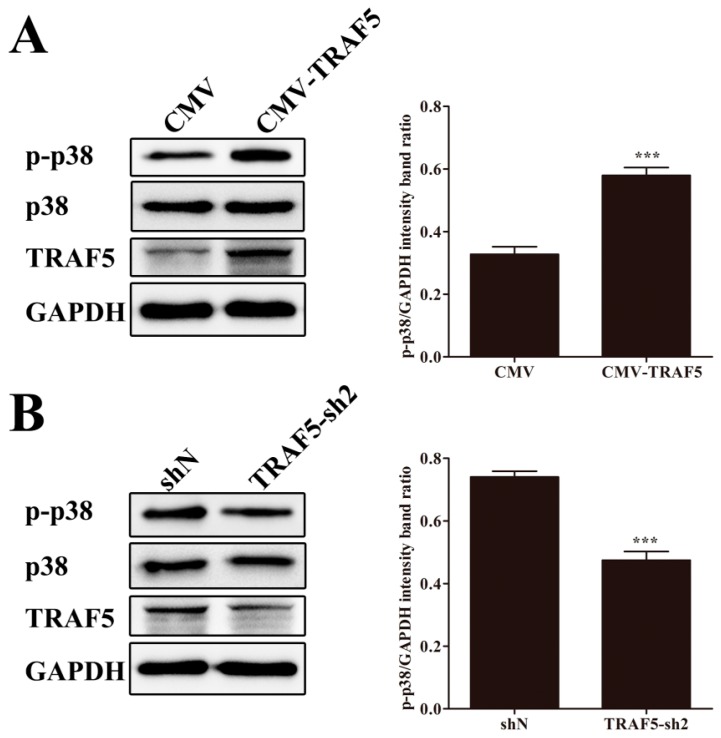
TRAF5 promotes p38 MAPK activation in PAMs. (**A**) TRAF5 overexpression promotes p38 MAPK activation. The cell lysates were collected in CMV and CMV-TRAF5 cells, and p-p38, p38 and TRAF5 expression was detected by Western blot; (**B**) TRAF5 knockdown reduces p38 MAPK activation. The shN and TRAF5-sh2 cells were infected with CSFV for 24 h, and p-p38, p38 and TRAF5 expressions were detected by Western blot. The relative levels of the p-p38 protein were estimated by histograms representing density readings of the gel bands, and the ratios were calculated relative to GAPDH control. The data represent the mean ± SD of three independent experiments. ***, *p* < 0.001.

Having validated the activation of p38 MAPK during CSFV infection, we investigated the effect of TRAF5 on p38 MAPK activation. In stable TRAF5-overexpressing cells, we observed higher levels of p-p38 and unchanged p38 levels (Figure 5A). In CSFV-infected stable TRAF5-knockdown cells, the p-p38 level was reduced compared with that in CSFV-infected shN cells (Figure 5B), indicating that TRAF5 knockdown reduced p38 MAPK activation induced by CSFV. These data demonstrated that TRAF5 promotes p38 MAPK activation in PAMs.

### 3.7. Inhibition of p38 MAPK Activation Suppresses CSFV Replication in PAMs

**Figure 6:**
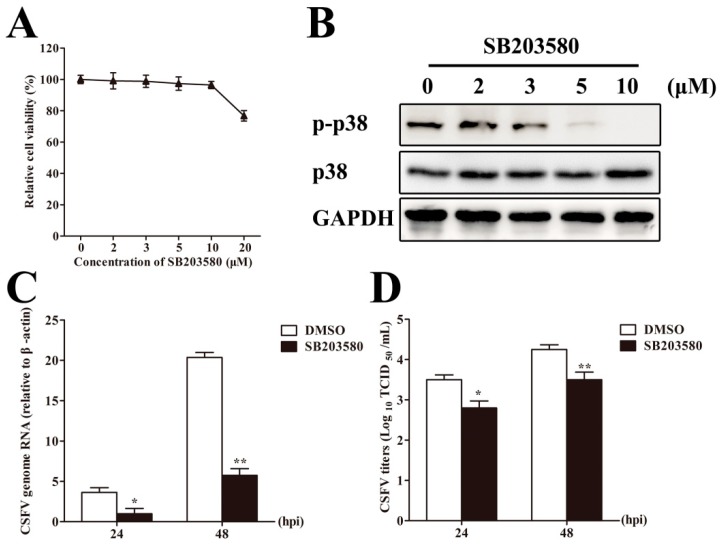
Inhibition of p38 MAPK activation suppresses CSFV replication in PAMs. (**A**) The cell viability in the SB203580 treatment. PAMs were treated with different concentrations of SB203580 for 1 h; then, the culture medium was replaced with fresh medium for 48 h and the cell viability was measured by the CCK-8 assay; (**B**) The effect of SB203580 on p38 MAPK activation. PAMs were treated with SB203580 at a concentration of 0, 2, 3, 5, or 10 μM for 1 h, followed by replacement with flesh medium and inoculation with CSFV at an MOI of 0.1. At 24 hpi, the cell lysates were subjected to Western blot to determine p-p38 and p38 expression; (**C**,**D**) SB203580 treatment suppresses CSFV replication in PAMs. PAMs were treated with SB203580 at a concentration of 10 μM for 1 h; DMSO treatment as a negative control, followed by replacement with flesh medium and inoculation with CSFV at an MOI of 0.1. CSFV genome RNA in the cell lysates (**C**) was analyzed by RT-qPCR, and viral titers in the supernatant (**D**) were assessed and expressed as TCID_50_/mL at 24 and 48 hpi. The data represent the mean ± SD of three independent experiments. *, *p* < 0.05; **, *p* < 0.01.

To confirm whether p38 MAPK is involved in the regulation of CSFV replication, we detected the CSFV genome RNA and viral titers in CSFV-infected PAMs with p38 MAPK inhibitor SB203580 treatment. DMSO treatment was used as a control. Firstly, the optimal concentration of SB203580 was selected. The results showed that the concentration of SB203580 of up to 10 μM had no effects on cell viability (Figure 6A), and SB203580 at 10 μM completely inhibited p38 MAPK activation following CSFV infection (Figure 6B). Thus, the concentration of SB203580 of 10 μM was used in the following experiments. In SB203580-treated CSFV-infected PAMs, CSFV genome RNA (Figure 6C) and viral titers in the supernatant (Figure 6D) were decreased compared with those in DMSO-treated PAMs at 24 and 48 hpi. The results indicate that inhibition of p38 MAPK activation suppresses CSFV replication.

### 3.8. TRAF5 Does not Promote CSFV Replication Following Inhibition of p38 MAPK Activation

**Figure 7:**
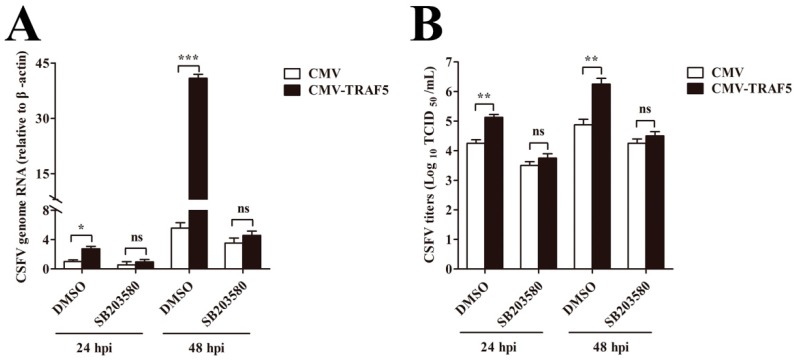
TRAF5 overexpression does not promote CSFV replication following inhibition of p38 MAPK activation in PAMs. (**A**,**B**) CSFV replication in SB203580-treated CMV-TRAF5 cells. CMV and CMV-TRAF5 cells were treated with SB203580 for 1 h, followed by CSFV infection. The cell lysates and the culture supernatant were subjected to determine the CSFV genome RNA (**A**) and viral titers (**B**) at 24 and 48 hpi, respectively. The data represent the mean ± SD of three independent experiments. *, *p* < 0.05; **, *p* < 0.01; ***, *p* < 0.001; ns: not significant.

Having demonstrating that TRAF5 promotes CSFV replication and p38 MAPK activation and that p38 MAPK activation is involved in CSFV propagation, we investigated whether TRAF5 promoted CSFV replication via p38 MAPK activation. In CMV and CMV-TRAF5 cells, we blocked p38 MAPK activation by SB203580 and examined CSFV replication at 24 and 48 hpi. After blocking the pathway, TRAF5 overexpression failed to promote viral genome RNA (Figure 7A) and viral titers (Figure 7B), indicating that TRAF5 promotes CSFV replication via p38 MAPK activation.

## 4. Discussion

CSFV NS3 is a multifunctional non-structural protein that plays a major role in CSFV replication [5,32,33]. Our previous study reported 26 interacting partners of CSFV NS3 (including TRAF5) by Y2H screening, and TRAF6 was verified to interact with NS3 and inhibit CSFV replication [10]. However, the roles of other proteins interacting with NS3 during CSFV infection have not been reported. Here, we further investigated the role of TRAF5 in CSFV replication. We first cloned the full-length of TRAF5 and NS3 to verify the TRAF5-NS3 interaction by the Y2H approach. In addition, co-IP and GST pull-down assays were performed to further confirm the true TRAF5-NS3 interaction. However, the interaction domain of NS3 and TRAF5 and whether NS3 interacts with other TRAFs will be further investigated in future studies.

TRAF6 interacts with CSFV NS3 and inhibits CSFV replication via NF-κB signaling pathway [10]. uS10 interacts with CSFV N^pro^ and inhibits CSFV replication via regulating TLR3 expression [30]. In addition, Rab1A interacts with CSFV NS5A and is required for the assembly of CSFV particles [34]. Rab5 interacts with CSFV NS4B and enhances CSFV proliferation [35]. Here, we demonstrated that TRAF5 interacted with NS3 and promoted CSFV replication. In addition, CSFV and NS3 increased TRAF5 expression, indicating the synergistic effect of TRAF5 and CSFV, and the potential role of TRAF5 in CSFV replication capacity. Similarly, it has been reported that TRAF5 interacts with the HIV-1 Nef protein, and HIV-1 Nef activates TRAF5 to increase HIV-1 replication in monocyte-derived macrophages (MDMs) [36]. These results offer an insight into the mechanisms related to CSFV infection via TRAF5 upregulation by CSFV NS3.

TRAF5 is an intracellular protein that binds to the cytoplasmic portion of TNF receptors and mediates the activation of downstream NF-κB, IRF3 and MAPK signaling pathways, controlling cellular processes as well as immune responses [15,16]. TRAF5 negatively regulates toll-like receptor signaling in B lymphocytes [13]. It has been reported that CSFV infection activates ERK1/2, and ERK1/2 activation enhances CSFV replication [37,38]. Hepatitis C virus (HCV), belonging to the family *Flaviviridae*, activates p38 MAPK during HCV replication [39,40]. Here, we first demonstrated that CSFV infection and TRAF5 expression activated p38 MAPK in PAMs. Similarly, TRAF5 expression induced p38 MAPK activation in HeLa cells [30], and GITR-induced stimulation of TRAF5-deficient T cells resulted in decreased activation of p38 MAPK [27]. Whether CSFV NS3 promotes p38 MAPK activation or whether CSFV infection activates c-Jun N-terminal kinase (another family of MAPK) needs further study; however, these data demonstrate that CSFV infection activates p38 MAPK and that TRAF5 may function as a link between CSFV infection and the p38 MAPK activation.

p38 MAPK is involved in the regulation of inflammatory cytokine expression and is an inducible NO synthase, and the inhibitors of the p38 MAPK signaling pathway are severed as candidate anti-inflammatory drugs [41]. Here, we demonstrated that inhibition of p38 MAPK activation by SB203580 severely suppressed CSFV replication in PAMs, indicating that p38 MAPK is involved in CSFV efficient replication and SB203580-derived agents may be used for potential antivirals against CSFV infection. TRAF5 activation negatively regulates the latent replication origin of the Epstein-Barr virus through p38 MAPK [28]. TRAF5 induces HIV-1 gene expression via activation of p38 MAPK in monocytes/macrophages [29]. Notably, we demonstrated that TRAF5 did not promote CSFV replication following inhibition of p38 MAPK activation, indicating a possible mechanism that TRAF5 activates p38 MAPK to increase CSFV replication.

p38 MAPK is involved in interleukin-10 (IL-10) production induced by porcine reproductive and respiratory syndrome virus (PRRSV) and porcine circovirus type 2 in PAMs [42,43]. IL-10, an important anti-inflammatory and immunosuppressive cytokine, inhibits cell-mediated immune responses and antigen presentation [44,45]. Many viruses induce IL-10 production, and certain viruses encode a viral IL-10 homologue to establish infection using the immunosuppressive properties of IL-10 [46]. For example, IL-10 is produced constitutively during HIV-1 infection, and is considered as an important pathway by which HIV may induce immunodeficiency [47]. In addition, foot-and-mouth disease virus (FMDV) induces IL-10 production to mediate immunosuppression during acute FMDV infection in porcine DCs [48]. Moreover, CSFV infection induces IL-10 expression [37], and CSFV vaccination is immunosuppressed in the presence of the PRRSV vaccine, which may be due to the fact that PRRSV vaccination induces higher levels of IL-10 expression [49]. Thus, we speculate that TRAF5 may regulate IL-10 expression by activating p38 MAPK to suppress the immune response during CSFV infection and to promote CSFV replication. However, this speculation needs further investigation.

In conclusion, we show that cellular TRAF5 interacts with CSFV NS3 and enhances CSFV replication via activation of p38 MAPK. Further studies are needed to elucidate the precise molecular mechanisms of how p38 MAPK activated by TRAF5 enhances CSFV replication. However, these findings provide new insights into the mechanisms of CSFV infection, as well as potential antiviral strategies focused on attenuating CSFV replication.

## Figures and Tables

**Table 1:** Primers used in this study.

Primers	Sequence (5′ → 3′)	Purpose
BD-NS3-F	GGAATTCCATATGCCTAAGAAAAAGCGCAAAGTTGGGCCTGCCGTTTGCAAGAAG	Amplification of NS3
BD-NS3-R	AACTGCAGTAGCTCCTTCAATTCTGTCTCCTTCCCCTC	
NS3-GFP-F	CATGCTAGCGATGGGGCCTGCCGTTTGCAAGAAG	Amplification of NS3
NS3-GFP-R	AACTGCAGTAGACCAACTACTTGTTTTAGTGCTCTGCC	
CMV-NS3-F	CTAGCTAGCATGGGGCCTGCCGTTTGC	Amplification of NS3
CMV-NS3-R	ATAAGAATGCGGCCGCTTACTTATCGTCGTCATCCTTGTAATCTAGACCAACTACTTG	
GST-NS3-F	ACGCGTCGACTCGGGCCTGCCGTTTGCA	Amplification of NS3
GST-NS3-R	ATAAGAATGCGGCCGCTCATAGACCAACTACTTGTTTTAGTGC	
RFP-F	GGAATTCATGGTGCGCTCCTCCAAGAAC	Amplification of RFP
RFP-R	CGGGATCCCTACTTATCGTCGTCATCCTTGTAATCCAGGAACAGGTGGTGGCGG	
AD-TRAF5-F	GGAATTCCATATGATGGCCTCTTCTGAGGAGCAAG	Amplification of TRAF5
AD-TRAF5-R	CGGGATCCTTAGAGATCCTCCAGGTCAGTTAAATCC	
CMV-TRAF5-F	GCTCTAGAATGGCCTCTTCTGAGGAGCAAG	Amplification of TRAF5
CMV-TRAF5-R	CGGGATCCGAGATCCTCCAGGTCAGTTAAATCC	
GST-TRAF5-F	CGGGATCCATGGCCTCTTCTGAGGAGCAAG	Amplification of TRAF5
GST-TRAF5-R	CCGCTCGAGTTAGAGATCCTCCAGGTCAGTTAAATCC	
β-actin-F	CAAGGACCTCTACGCCAACAC	RT-qPCR for detection of β-actin
β-actin-R	TGGAGGCGCGATGATCTT	
CSFV-F	GATCCTCATACTGCCCACTTAC	RT-qPCR for detection of CSFV RT-qPCR for detection of TRAF5
CSFV-R	GTATACCCCTTCACCAGCTTG	
TRAF5-F	GGGGAGACTAACAAACATGATG	
TRAF5-R	GTAGAAGGGCTGGCTGAAGA	
TRAF5-sh1-F	GATCCGGTGTACGGCCAAGATCATTCTCAAGAGGAATGATCTTGGCCGTACACCTTTTTG	Knockdown of TRAF5
TRAF5-sh1-R	AATTCAAAAAGGTGTACGGCCAAGATCATTCCTCTTGAGAATGATCTTGGCCGTACACCG	
TRAF5-sh2-F	GATCCGGAAGGTGACAGACTACAAGCTCAAGAGGCTTGTAGTCTGTCACCTTCCTTTTTG	Knockdown of TRAF5
TRAF5-sh2-R	AATTCAAAAAGGAAGGTGACAGACTACAAGCCTCTTGAGCTTGTAGTCTGTCACCTTCCG	
TRAF5-sh3-F	GATCCGCACCTGTCGCTGTACTTTGTTCAAGAGACAAAGTACAGCGACAGGTGCTTTTTG	Knockdown of TRAF5
TRAF5-sh3-R	AATTCAAAAAGCACCTGTCGCTGTACTTTGTCTCTTGAACAAAGTACAGCGACAGGTGCG	

Restriction enzyme sequences or silence sequences underlined.
